# DNA methylation signatures of long intergenic noncoding RNAs in porcine adipose and muscle tissues

**DOI:** 10.1038/srep15435

**Published:** 2015-10-23

**Authors:** Zhong-Yin Zhou, Aimin Li, Li-Gang Wang, David M Irwin, Yan-Hu Liu, Dan Xu, Xu-Man Han, Lu Wang, Shi-Fang Wu, Li-Xian Wang, Hai-Bing Xie, Ya-Ping Zhang

**Affiliations:** 1 State Key Laboratory of Genetic Resources and Evolution, and Yunnan Laboratory of Molecular Biology of Domestic Animals, Kunming Institute of Zoology, Chinese Academy of Sciences, Kunming, China; 2 Department of Molecular and Cell Biology, School of Life Sciences, University of Science and Technology of China, Hefei, China; 3 School of Computer Science and Engineering, Xi’an University of Technology, Xi’an, China; 4 Key Laboratory of Farm Animal Genetic Resources and Germplasm Innovation of Ministry of Agriculture, Institute of Animal Science, Chinese Academy of Agricultural Sciences, Beijing, China; 5 Department of Laboratory Medicine and Pathobiology, University of Toronto, Toronto, Canada; 6 Banting and Best Diabetes Centre, University of Toronto, Toronto, Canada; 7 Laboratory for Conservation and Utilization of Bio-resources, Yunnan University, Kunming, China; 8 Kunming College of Life Science, University of Chinese Academy of Sciences, Kunming, China

## Abstract

Long intergenic noncoding RNAs (lincRNAs) are one of the major unexplored components of genomes. Here we re-analyzed a published methylated DNA immunoprecipitation sequencing (MeDIP-seq) dataset to characterize the DNA methylation pattern of pig lincRNA genes in adipose and muscle tissues. Our study showed that the methylation level of lincRNA genes was higher than that of mRNA genes, with similar trends observed in comparisons of the promoter, exon or intron regions. Different methylation pattern were observed across the transcription start sites (TSS) of lincRNA and protein-coding genes. Furthermore, an overlap was observed between many lincRNA genes and differentially methylated regions (DMRs) identified among different breeds of pigs, which show different fat contents, sexes and anatomic locations of tissues. We identify a lincRNA gene, linc-sscg3623, that displayed differential methylation levels in backfat between Min and Large White pigs at 60 and 120 days of age. We found that a demethylation process occurred between days 150 and 180 in the Min and Large White pigs, which was followed by remethylation between days 180 and 210. These results contribute to our understanding of the domestication of domestic animals and identify lincRNA genes involved in adipogenesis and muscle development.

Long noncoding RNAs (lncRNAs) are a class of transcripts that are longer than 200 nt in length and do not encode proteins. Similar to mRNAs, lncRNAs are transcribed by RNA polymerase II and undergo splicing and polyadenylation. LncRNAs can be classified into antisense transcripts, long intronic noncoding RNAs and long intergenic noncoding RNAs (lincRNAs), according to their position relative to protein-coding genes. Some lincRNAs have been indicated to play important roles in a variety of biological processes, such as dosage compensation^1^,^2^,^3^,^4^, transcriptional regulation^5^,^6^,^7^, epigenetic regulation^8^,^9^ and pluripotency maintenance^10^. Previous studies have demonstrated that lincRNAs play a role in adipogenesis^11^,^12^ and muscle development^13^.

The pig is an emerging medical model for studying energy metabolism and obesity in humans, since both possess similar cardiovascular systems, metabolic features, and proportional organ size^14^. Thousands of years of selection on pigs have created abundant phenotypic variation, for example, different pig breeds show varying performance in adipose and lean meat production. Therefore, these breeds should be valuable models for studies of adipogenesis and muscle development.

In an earlier study, Li *et al*. performed an investigation on DNA methylation in eight different adipose and two distinct skeletal muscle tissues from three breeds of pig^15^, where they found differentially methylated regions in the promoters of the protein-coding genes are highly associated with the development of obesity^15^. In a different study, we identified 6,621 lincRNAs encoded by 4,515 gene loci in the pig genome^16^. Combining data from these two studies provides an opportunity to study the DNA methylation of lincRNAs loci in adipose and muscle tissues.

Here, we investigated the genome-wide levels of DNA methylation for lincRNA genes in adipose and muscle tissues from three breeds: Landrace, Rongchang and Tibetan. Comparison of the methylation patterns observed in protein-coding and lincRNA genes identified several distinctive methylation characteristics that differ between these classes of genes. We also analyzed differentially methylation regions (DMRs) that overlap lincRNA genes. This study contributes to our understanding of the DNA methylation of lincRNA genes and provides a valuable resource for the functional studies of lincRNAs that are associated with adipogenesis and muscle development.

## Result

### Global patterns of DNA methylation in lncRNA genes

**Figure 1:**
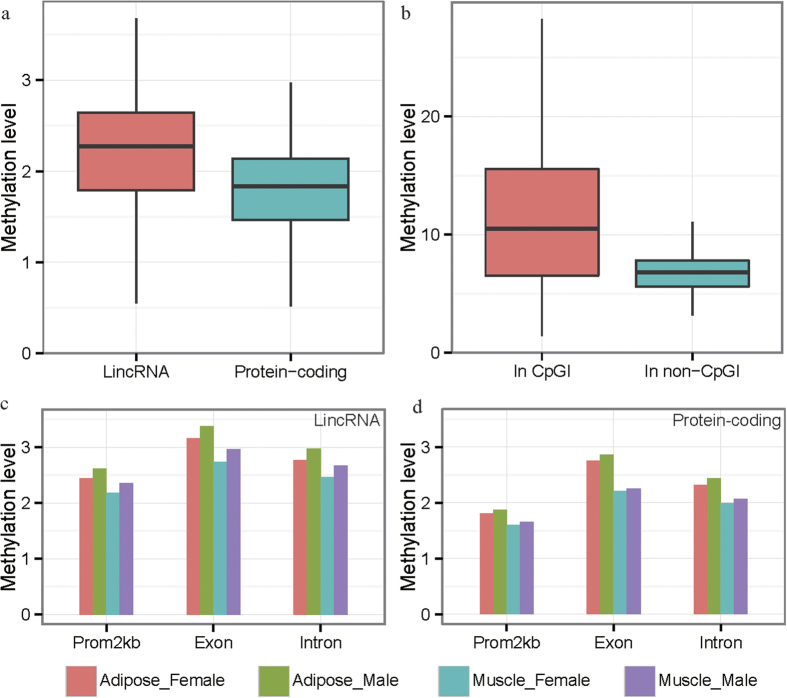
DNA methylation levels in lincRNA and protein-coding genes. (**a**) Box plots for the comparison of DNA methylation levels between lincRNA and protein-coding genes. (**b**) Methylation levels of CpG sites in CpGIs and non-CpGIs for lincRNA genes. (**c**) Average methylation levels within the promoters, exons, and introns of lincRNA genes. Different bins represent adipose in females (Adipose_Female), and males (Adipose_Male), and muscle in females (Muscle_Female) and males (Muscle_Male). (**d**) Average methylation levels within the promoters, exons, and introns of protein-coding genes. Bins are defined as in (**c**). Methylation levels were normalized for read depth using an overall average amount of reads form the 180 samples.

In our previous study, we found pig lincRNA genes have several characteristics which differ from those of mRNA genes such as their length, number of exons and level of expression^16^. Here, we found the GC content (0.37) and observed-over-expected number of CpG (CpG_o/e_) ratio (0.26) of lincRNA genes are similar to those of protein-coding genes (GC content: 0.38, CpG_o/e_: 0.29) in the genomic regions that span from 2 kb upstream of the transcription start sites (TSS) to the transcription end site (TES) for both of these types of genes. However, the methylation levels of the lincRNA genomic regions were significantly higher than that for the mRNA genes (Kolmogorov-Smirnov test, P = 2.621 × 10^−10^, Fig. 1a), which indicates that there is a differential methylation pattern between protein-coding and lincRNA genes. The results may be due to differential methylation regulation mechanisms between lincRNA and protein-coding genes.

Among the 768,645 CpG sites in the lincRNA genomic region (−2K upstream TSS to TES) dataset, 85,012 CpG sites are located in CpGIs (CpG islands) and the remaining 683,633 CpG sites are not in CpGIs (non-CpGIs). Differing from the previous result of a microarray study^17^, the average DNA methylation level across all samples at CpGs in CpGIs was significantly higher those of non-CpGIs (Kolmogorov-Smirnov test, p < 2.2 × 10^−16^, Fig. 1b).

Methylation levels of exons, introns and promoters were compared between lincRNA and protein-coding genes for adipose and muscle tissues of both female and male pigs. Our analysis found that the methylation levels of exons, introns and promoters of the lincRNA genes were always higher than those of mRNA genes (Kolmogorov-Smirnov test, P < 2.2 × 10^–16^, Supplementary Fig. S1 and Fig. 1c,d). Consistent with Sati *et al*.^18^, we found that the methylation pattern of exons, introns and promoters of lncRNA genes were similar to those of protein-coding genes, with exons having higher methylation levels than introns or promoters (Fig. 1c,d).

### The patterns of DNA methylation across TSS of lincRNA genes

**Figure 2:**
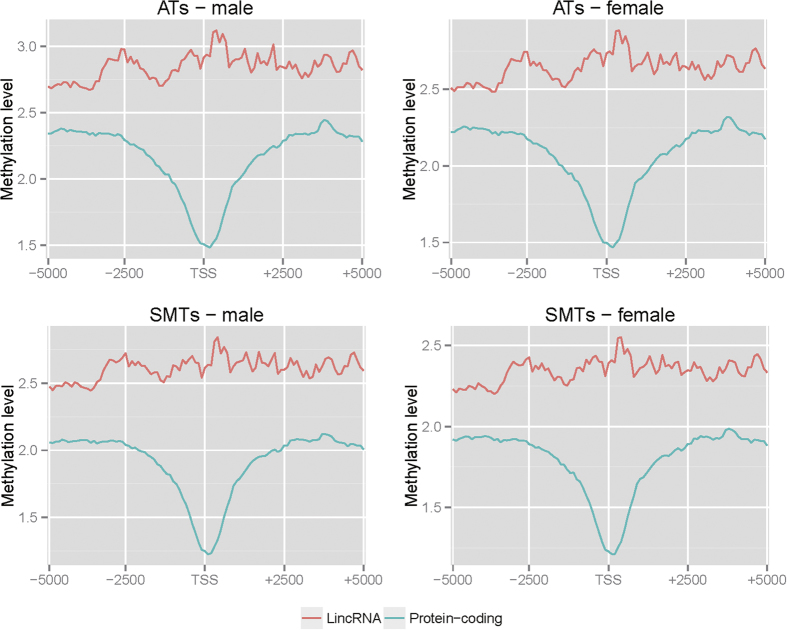
DNA methylation patterns around the TSS of lincRNA and protein-coding genes. Distribution of the methylation level was calculated in 100-bp sliding windows, 5-kb upstream and downstream from the TSS. ATs–male: male adipose tissues, ATs–female: female adipose tissues, SMTs–male: male skeletal muscle tissues, SMTs–female: female skeletal muscle tissues.

In humans, the methylation pattern across the TSS of lincRNAs is different from that of protein-coding genes^18^. In pigs, we found similar methylation patterns in male and female, for both adipose and muscle tissues, with the methylation level across the TSS of lincRNA genes being higher than for protein-coding genes (Fig. 2). The TSS of protein-coding genes showed a V-shaped curve for methylation level indicating a relative lowing of the methylation density (Fig. 2), in concordance with a previous report^18^. In contrast to mRNA genes, we found a slightly increased methylation level around the TSS of lncRNA genes (Fig. 2). In an earlier study^18^, Sati *et al*. found a sharp peak immediately downstream of the TSS of lincRNA genes in humans, whereas no similar results were found for the pig genes in this study. These observations indicate a different pattern of methylation occurs around the TSS of lincRNA and protein-coding genes. In this study, only lincRNA genes that are located at least 500bp away from a protein-coding or a house-keeping gene were included, to reduce the influence due to potential overlap between the exons of a lincRNA and a protein-coding gene.

**Figure 3:**
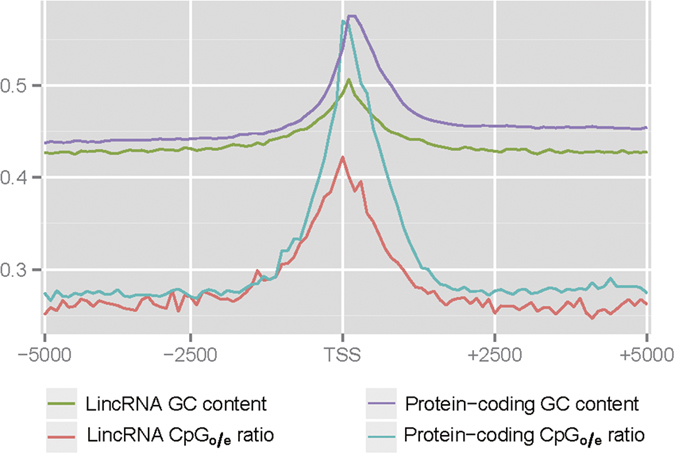
Distribution of the GC content and CpG_o/e_ ratio around the TSS of lincRNA and protein-coding genes. Distributions were calculated in 100-bp sliding windows, 5-kb upstream and downstream from the TSS.

GC content and CpG_o/e_ across TSSs were compared between pig lincRNA and protein-coding genes. Both lincRNA and protein-coding genes showed higher GC content and CpG_o/e_ near their TSSs (Fig. 3). When this result is combined with our observations on the methylation levels around TSS, we concluded that the differential methylation pattern seen between lincRNA and mRNA genes is most likely due to differential regulation of DNA methylation, rather than nucleotide composition. The above observations could also partly explain why the majority of lincRNA genes show tissue-specific and developmental stage-specific expression.

### DMRs located in lincRNA genes

The study by Li *et al*. on genome-wide DNA methylation levels was performed on three breeds of pigs (Landrace, Rongchang and Tibetan) that show different obesity and muscle-related phenotypes^15^. To study the regulation of adipocytes and muscle development, they adipose tissue (AT) was sampled from 8 diverse anatomic locations as well as two skeletal muscle tissues (SMT), white longissimus dorsi muscle (LDM) and red psoas major muscle (PMM)^15^. Phenotypic differences were seen in the sampled adipose and skeletal muscle tissues between breeds, sexes and anatomic locations^15^.

To identify lincRNA genes associated with adipocyte regulation and muscle development, we re-analyzed the methylation datasets from Li *et al*.^15^ using MEDIPS package^19^ and found a considerable number of differentially methylated regions (DMRs) that overlap with lincRNA gene regions (Table 1).

Interestingly, we found that *Xist*, a lncRNA associated with X chromosome inactivation^2^, is located in a DMR between sexes for adipose. DMRs in lincRNA gene regions have a higher GC content (0.47) and CpG_o/e_ ratios (0.39) than the average for lincRNA genes. A total of 4,139 DMRs were found to be located in the promoter regions of lincRNA genes. We then classified lincRNA promoters into three classes: HCPs (high-CpG promoters), ICPs (intermediate CpG promoters) and LCPs (low-CpGs promoters) according to their CpG profiles as previously described^20^. Similar to protein-coding genes^15^, DMRs of lincRNA genes are most frequently located in ICP than in HCP or LCP (hypergeometric test, P < 2.2 × 10^−16^). Consistent with previous studies^15^,^21^,^22^,^23^, we found that most DMRs associated with lncRNA genes are located in CpGIs rather than non-CpGIs (hypergeometric test, P < 2.2 × 10^−16^).

### Candidate lincRNA genes associated with adipogenesis in the pig

**Figure 4:**
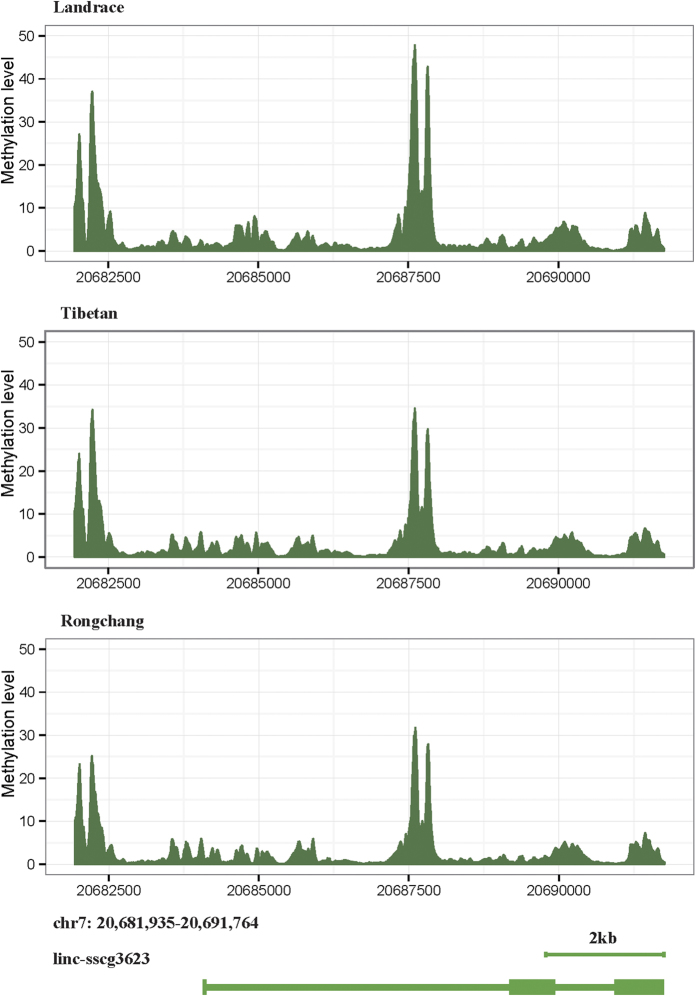
Comparison of the methylation levels for eight adipose tissues from differential body sites of Rongchang, Tibetan and Landrace breeds for linc-sscg3623 gene.

In eight adipose tissues from different body sites, linc-sscg3623 shows differences in level of methylation (located in DMRs) between the Landrace and Rongchang breed of 210-day-old pigs, with the methylation level of linc-sscg3623 being higher in Landrace than in Rongchang breed (Fig. 4), which is consistent with the difference in fat deposition between these two breeds^15^. As the Fig. 1a in Li *et al*.^15^, the ability to deposit fat is Rongchang>Tibetan>Landrace, which is contrary to the methylation level of linc-sscg3623 among the three breeds: Rongchang<Tibetan<Landrace (Fig.4).

### Dynamics of DNA methylation of lincRNA gene in backfat tissue

**Figure 5:**
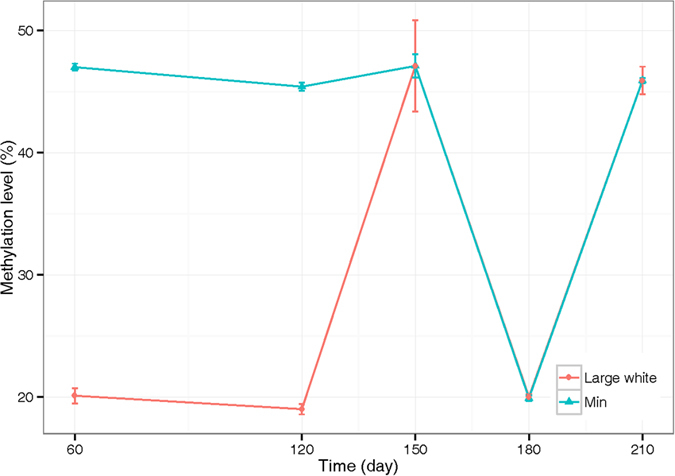
Comparison of the methylation levels for the GC site (chr7:20684724) in linc-sscg3623 gene from genomic DNA from backfat tissue at different developmental stages in Min and Large White breeds of pig.

To further investigate if the linc-sscg3623 gene is involved in adipogenesis, we used bisulfite pyrosequencing to investigate the methylation level of two GC sites (located in DMRs) in this gene in backfat from five developmental stages (60, 120, 150, 180 and 210 days) in Min and Large White pigs. Min pigs have a higher methylation level than the Large White pigs at 60 and 120 days, which is consistent with the differential fat deposition between the two breeds (Fig. 5, Supplementary Fig. S2 and Table S2). At 150 days and later, similar methylation patterns are observed between the Min and Large White pigs (Fig. 5, Supplementary Fig. S2 and Table S2). The data suggest a demethylation process occurred between days 150 and 180 in both pigs, which was followed by a remethylation process between days 180 and 210 (Fig. 5, Supplementary Fig. S2 and Table S2). The demethylation and remethylation processes may be associated with adipocyte differentiation. At day 210, we could not find any difference in the methylation level between the Min and Large White breeds for backfat tissue. This may be due to differential fat deposition mechanisms in differential adipose sites. These observations suggest that linc-sscg3623 may show differences in expression level between differential breeds or development stages and affect adipogenesis.

## Discussion

Several studies have indicated that DNA methylation plays important roles in stem cell differentiation^24^ and embryonic development^25^, and that alternations in DNA methylation are associated with disease^26^. These studies, though, mainly focused on protein-coding genes. In this study, we characterized the DNA methylation patterns of pig lincRNA genes in adipose and muscle tissues.

To compare DNA methylation pattern between lincRNA and protein-coding genes, we focused on the genomic regions consisting of their promoters and their gene bodies, and found that lincRNA genes have higher DNA methylation levels than protein-coding genes despite lincRNA genes having similar GC content and CpG_o/e_ as protein-coding genes. When we considered promoters, exons or introns separately, the level of methylation for lincRNA genes is higher for each region compared to the corresponding parts of the protein-coding genes. We also found that the methylation level of CpG sites in CpGIs was higher than for non-CpGIs for lincRNA genes, in contrast to previous studies of genomic CpG sites^17^, which indicated that the methylation levels of CpG sites in CpGIs was lower than that of non-CpGIs. GC content and CpG_o/e_ across TSS showed similar patterns between protein-coding and lincRNA genes, whereas the methylation levels across TSS of these genes display differences. These features imply that DNA methylation is differentially regulated between lincRNA and protein-coding genes and may partly explain why the expression level of lincRNA genes is lower than that of protein-coding genes.

In this study, we only focused on the pattern of methylation of lincRNA genes in adipose and muscle tissues of pigs, and these results should contribute to our understanding of the roles of lincRNAs in tissue-specific regulatory mechanisms, including those used in humans and mouse. In our previous studies of pig lincRNA genes, we indicated that these genes might have contributed to the domestication of the pig^16^. Fatness and lean muscle growth are two phenotypes that have experienced strong artificial selection in pigs. Here, we found many lincRNA genes are located in DMRs, with many of the DMRs overlapping with the promoters of these genes. Recently, a limited number of lincRNAs were identified to be involved in adipogenesis^11^,^12^. Interestingly, many lincRNA genes were found to overlap with DMRs. For example, linc-sscg3623 showed different methylation levels in adipose tissues between the Landrace and Rongchang breeds of pigs that differ in fatness. The Min and Large White breeds also showed different methylation levels in backfat tissues at two developmental stages, 60 and 120 days, and then displayed a demethylation process and a remethylation process between days 150 and 210. These results imply that lincRNA genes contribute to fatness and lean growth in pigs, and specific alleles may have been selected in different breeds. It is believed that DNA methylation in promoters is one of the regulatory mechanisms that influence gene expression. Li *et al*. had used a gene expression microarray to measure the expression levels of genes^15^, however their microarray contained only a small number of lincRNA genes (data not shown), thus, it is impossible for us to calculate any correlations between methylation levels in lincRNA promoters and the expression levels of the associated lincRNA genes using the MeDIP-seq data they generated^15^. Additional studies are needed to investigate the mechanisms controlling DNA methylation in lincRNA gene promoters for regulating their gene expression.

In summary, we found several differences in the methylation patterns between lincRNA and protein-coding genes, including differences in methylation levels and the pattern of methylation around their TSS. These results provide avenues for more in-depth research into the methylation patterns of lincRNA genes. Furthermore, we identified many lincRNA genes that are overlapped with DMRs which may help uncover the molecular basis of adipogenesis and muscle development and the further our understanding of the domestication of domestic animals.

## Material and Methods

All experimental protocols were approved by the Kunming Institute of Zoology, Chinese Academy of Sciences and Institute of Animal Science of the Chinese Academy of Agricultural Sciences.

### Data used

The methylated DNA immunoprecipitation sequencing (MeDIP-seq) dataset, which sampled eight adipose and two muscle tissues from three pig breeds including 180 samples^15^, was downloaded from the NCBI GEO database (GSE30344). Raw sequence reads were filtered as Li *et al*.^15^ and were aligned to the Sus scrofa 10.2 genome sequence using bwa aln (version 0.7.8-r455) with default parameters^27^. Protein-coding gene annotation was downloaded from the Ensembl database (version 73). The lincRNA annotation that we used in this study was generated from our previous study^16^. Pig CpG island (CpGI) positions were retrieved from the UCSC Genome Browser for the pig 10.2 genome. CpGI shores are located within 2 kb of CpGIs.

### Definition of promoters

In this study, we defined genomic region from −2000 to the TSS as the promoter for 6,572 lincRNA transcripts. These promoters were classified into three types according to CpG ratio as in a previous study^20^. There are 1,100 HCPs, 2,711 ICPs and 2,761 LCPs for the lincRNA transcripts.

### Identification of DMRs

To identify DMRs among the different breeds, sexes and anatomic locations, we used edgeR integrated in the Bioconductor package MEDIPS at genome-wide 250 bp bins^19^. MEDIPS inferred differential methylation for the sample groups by calculating Wilcoxon rank tests for the reads per million (rpm) values of each window^19^. DMRs were filtered for windows with adjusted P < 0.1 (exact test for negative binomial distribution, using edgeR integrated in the Bioconductor package MEDIPS).

### Bisulfite pyrosequencing

Animals: Three biological replicates of 60, 120, 150, 180 and 210 day old Min and Large White pigs were used in this experiment (Supplementary Table S2). All animals were females and were fed under the identical normal conditions.

Tissue preparation: Animals were humanely killed in accordance with the guidelines of the Good Experimental Practices adopted by the Institute of Animal Science of the Chinese Academy of Agricultural Sciences. All backfat samples were collected between the third and fourth ribs, and were maintained in liquid nitrogen.

DNA methylation sequencing: Genomic DNA was isolated with the QIAamp DNA mini kit (Qiagen) and treated with bisulphite using EZ DNA methylation Gold kit (Zymo Research) according the manufacturer’s instructions. Detailed information regarding primer sequences is given in Supplementary Table S1. PCR amplification of interest regions was performed with a total reaction volume of 50 μl, using 10 μl 5Xbuffer (KAPA), 1 ul dNTP (10 mM/each), 1 ul forward primer (50pM/μl), 1 μl reverse primer (50pM/μl), 2 ul bisulfite-treated genomic DNA and water. PCR products were purified and sequenced by BGI Tech Solutions (Liuhe Beijing) Co., Limited using the PyroMark Q96 ID Pyrosequencing System (Qiagen). The methylation level was expressed as a percentage of the methylated cytosines over the sum of the methylated and unmethylated cytosines.

URLs. Differentially methylated regions (DMRs) identified in this study are online, http://res.xaut.edu.cn/aldb/download.html.

## Additional Information

**How to cite this article**: Zhou, Z.-Y. *et al*. DNA methylation signatures of long intergenic noncoding RNAs in porcine adipose and muscle tissues. *Sci. Rep*. **5**, 15435; doi: 10.1038/srep15435 (2015).

## Supplementary Material

Supplementary Information

## Figures and Tables

**Table 1:** Summary of differentially methylated regions (DMRs) identified by the MEDIPS software.

DMRs type	Number of DMRs	DMRs in lincRNA regions
Adipose S-DMRs (n = 72 per sex)	44,664	983
Muscle S-DMRs (n = 18 per sex)	4,861	109
Adipose T-DMRs (n = 18 per tissue)	163,995	1,203
Muscle T-DMRs (n = 18 per tissue)	491	65
Adipose B-DMRs (n = 48 per breed)	477,131	33,602
Muscle B-DMRs (n = 12 per breed)	415,929	28,304
Adipose (n = 144) versus muscle (n = 36) T-DMRs	108,361	9,316
