# Supplementary material for: DNA methylation signatures of long intergenic noncoding RNAs in porcine adipose and muscle tissues
Source: Sci Rep. 2015 Oct 23;5:15435. doi: 10.1038/srep15435 (PMC4616017; doi:10.1038/srep15435)
Supplement: Supplementary Information [file srep15435-s1.doc]

**Supplementary Information for**

**DNA methylation signatures of long intergenic noncoding RNAs in porcine adipose and muscle tissues**

Zhong-Yin Zhou,^1,2^ Aimin Li,^3^ Li-Gang Wang,^4^ David M Irwin,^1,5,6^ Yan-Hu Liu,^7^ Dan Xu,^1,8^ Xu-Man Han,^1^ Lu Wang,^7^ Shi-Fang Wu,^1^ Li-Xian Wang,^4*^ Hai-Bing Xie^1*^ and Ya-Ping Zhang^1,2,7*^

1 State Key Laboratory of Genetic Resources and Evolution, and Yunnan Laboratory of Molecular Biology of Domestic Animals, Kunming Institute of Zoology, Chinese Academy of Sciences, Kunming, China

2 Department of Molecular and Cell Biology, School of Life Sciences, University of Science and Technology of China, Hefei, China

3 School of Computer Science and Engineering, Xi’an University of Technology, Xi’an, China

4 Key Laboratory of Farm Animal Genetic Resources and Germplasm Innovation of Ministry of Agriculture, Institute of Animal Science, Chinese Academy of Agricultural Sciences, Beijing, China

5 Department of Laboratory Medicine and Pathobiology, University of Toronto, Toronto, Canada;

6 Banting and Best Diabetes Centre, University of Toronto, Toronto, Canada;

7 Laboratory for Conservation and Utilization of Bio-resources, Yunnan University, Kunming, China

8 Kunming College of Life Science, University of Chinese Academy of Sciences, Kunming, China

Authors for Correspondence: Dr. Ya-Ping Zhang (zhangyp@mail.kiz.ac.cn), Dr. Hai-Bing Xie (xiehb@mail.kiz.ac.cn), or Dr. Li-Xian Wang (iaswlx@263.net).

.

**Table of contents**

**Supplementary Fig. S1-S2**

Supplementary Fig. S1 | Comparison of the methylation levels between lincRNA and protein-coding genes in different gene elements (promoters, exons and introns), sex and tissues.

Supplementary Fig. S2 | Comparison of the methylation levels for backfat adipose tissues from different development stages of Min and Large White breeds of pig for the GC sites (chr7:20684732) in linc-sscg3623.

**Supplementary Tables S1-S2**

Supplementary Table S1 | Bisulfite pyrosequencing primer sequence information.

Supplementary Table S2 | Methylation levels of the GC sites for linc-sscg3623 gene using bisulfite pyrosequencing.


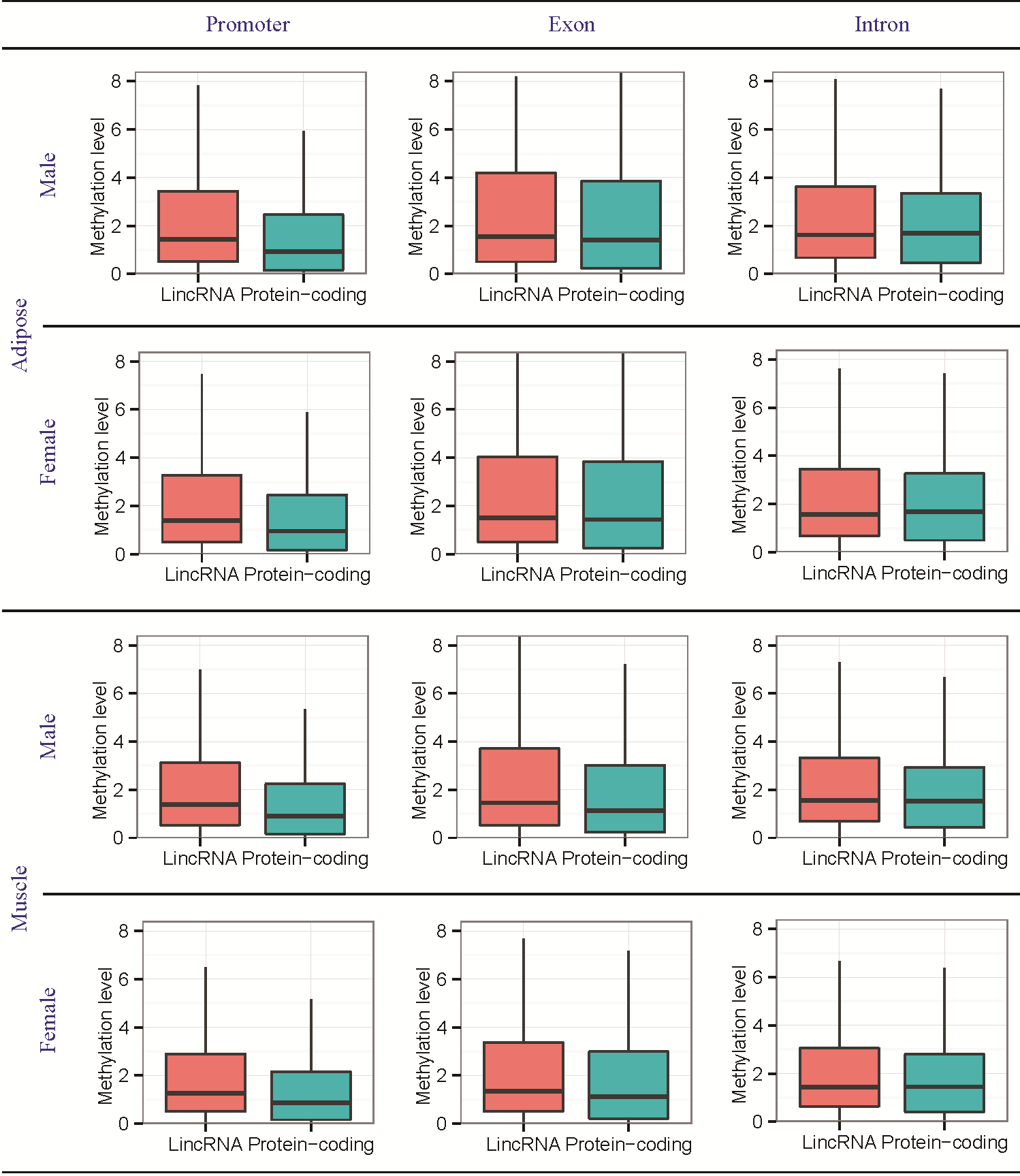


**Supplementary Fig. S1 | Comparison of the methylation level between lincRNA and protein-coding genes in different gene elements (promoters, exons and introns), sex and tissues.**


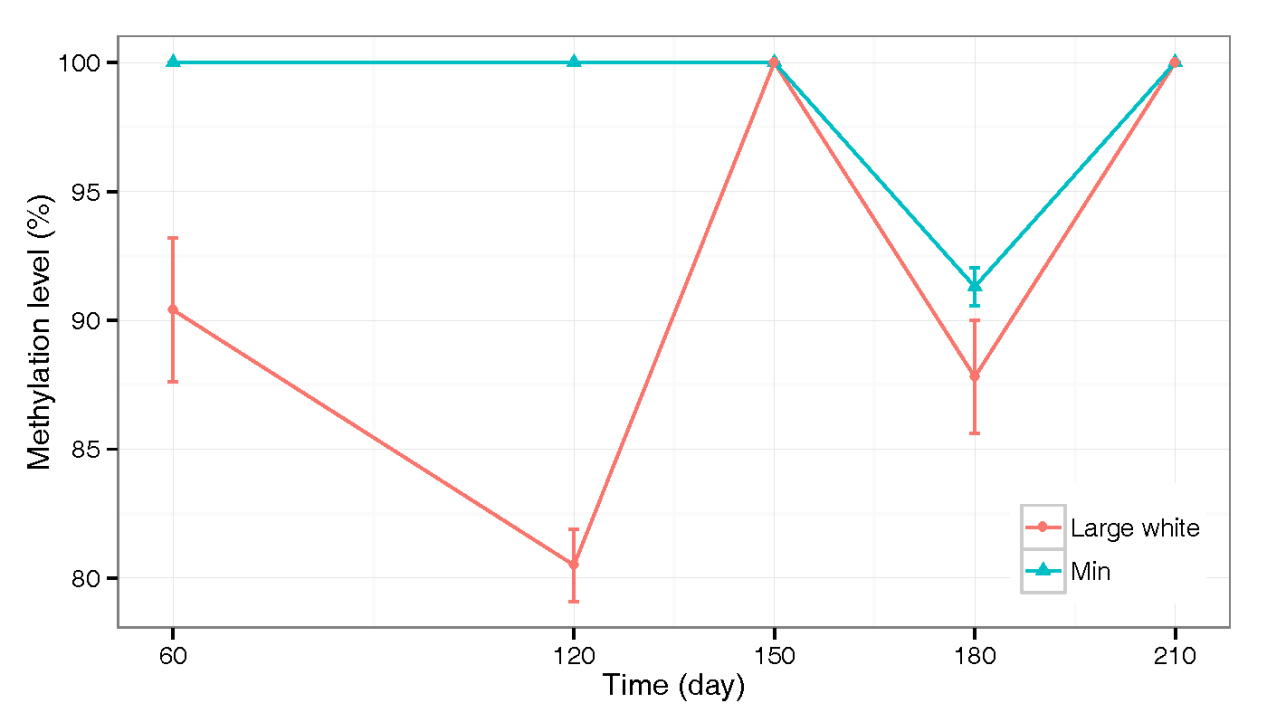


**Supplementary Fig. S2 |** **Comparison of the methylation levels for backfat adipose tissues from different development stage of Min and Large White breeds of pig for the GC sites (chr7:20684732) in linc-sscg3623.**

**Supplementary Table S1 | Bisulfite pyrosequencing primer sequence information**

| **Gene symbol** | **Primer set (Forward / Reverse / Sequence)** | **Target CpGs** |
| --- | --- | --- |
| *Linc-sscg3623* | F: 5’- TGGGTGAAGGATTTAAGTATTGTTG-3’ | 3 |
|  | R: 5’- AAAATTCCCCTAACTAAAAATCAAATCA -3’ |  |
|  | S: 5’- TGTGAGTTGTGGTGTA-3’ |  |

**Supplementary Table S2 | The methylation levels of the GC sites for linc-sscg3623 gene using bisulfite pyrosequencing.**

| Sample ID | Breed | Development Stages | Meth. (%)(chr7:20684724) | Meth. (%)(chr7:20684732) | Meth. (%) | Number of included CpGs | Mean |
| --- | --- | --- | --- | --- | --- | --- | --- |
| 1 | Min | 60days | 46.6 | 100 | 25.96 | 3 | 57.52 |
| 2 | Min | 60days | 47.19 | 100 | 31.55 | 3 | 59.58 |
| 3 | Min | 60days | 47.21 | 100 | 32.99 | 3 | 60.07 |
| 4 | Min | 120days | 45.2 | 100 | 15.97 | 3 | 53.72 |
| 5 | Min | 120days | 45.83 | 100 | 27.7 | 3 | 57.84 |
| 6 | Min | 120days | 45.1 | 100 | 27.11 | 3 | 57.4 |
| 7 | Min | 150days | 48.37 | 100 | 34.72 | 3 | 61.03 |
| 8 | Min | 150days | 46.1 | 100 | 29.53 | 3 | 58.54 |
| 9 | Min | 150days | 46.88 | 100 | 31.22 | 3 | 59.37 |
| 10 | Min | 180days | 19.97 | 90.75 | 22.42 | 3 | 44.38 |
| 11 | Min | 180days | 20.08 | 90.87 | 23.93 | 3 | 44.96 |
| 12 | Min | 180days | 19.53 | 92.38 | 22.32 | 3 | 44.74 |
| 13 | Min | 210days | 46.15 | 100 | 29.94 | 3 | 58.7 |
| 14 | Min | 210days | 45.68 | 100 | 31.27 | 3 | 58.98 |
| 15 | Min | 210days | 45.91 | 100 | 34.02 | 3 | 59.98 |
| 16 | Large White | 60days | 20.87 | 94.38 | 23.27 | 3 | 46.17 |
| 17 | Large White | 60days | 20.08 | 88.28 | 20.6 | 3 | 42.99 |
| 18 | Large White | 60days | 19.36 | 88.61 | 22.08 | 3 | 43.35 |
| 19 | Large White | 120days | 18.4 | 78.77 | 20.92 | 3 | 39.36 |
| 20 | Large White | 120days | 19.29 | 82.21 | 21.99 | 3 | 41.16 |
| 21 | Large White | 120days | 19.34 | 80.47 | 20.56 | 3 | 40.12 |
| 22 | Large White | 150days | 42.26 | 100 | 25.7 | 3 | 55.99 |
| 23 | Large White | 150days | 51.33 | 100 | 34.48 | 3 | 61.94 |
| 24 | Large White | 150days | 47.69 | 100 | 41.81 | 3 | 63.17 |
| 25 | Large White | 180days | 20.84 | 85.37 | 22.53 | 3 | 42.91 |
| 26 | Large White | 180days | 20.36 | 87.39 | 22.61 | 3 | 43.45 |
| 27 | Large White | 180days | 20.64 | 90.71 | 18.25 | 3 | 43.2 |
| 28 | Large White | 210days | 47.36 | 100 | 30.99 | 3 | 59.45 |
| 29 | Large White | 210days | 45.89 | 100 | 29.57 | 3 | 58.49 |
| 30 | Large White | 210days | 44.58 | 100 | 29.8 | 3 | 58.13 |
